# Supplementary figures and images for: Exploring the feasibility of technological visuo-cognitive training in Parkinson’s: Study protocol for a pilot randomised controlled trial
Source: PLoS One. 2022 Oct 7;17(10):e0275738. doi: 10.1371/journal.pone.0275738 (PMC9543984; doi:10.1371/journal.pone.0275738)

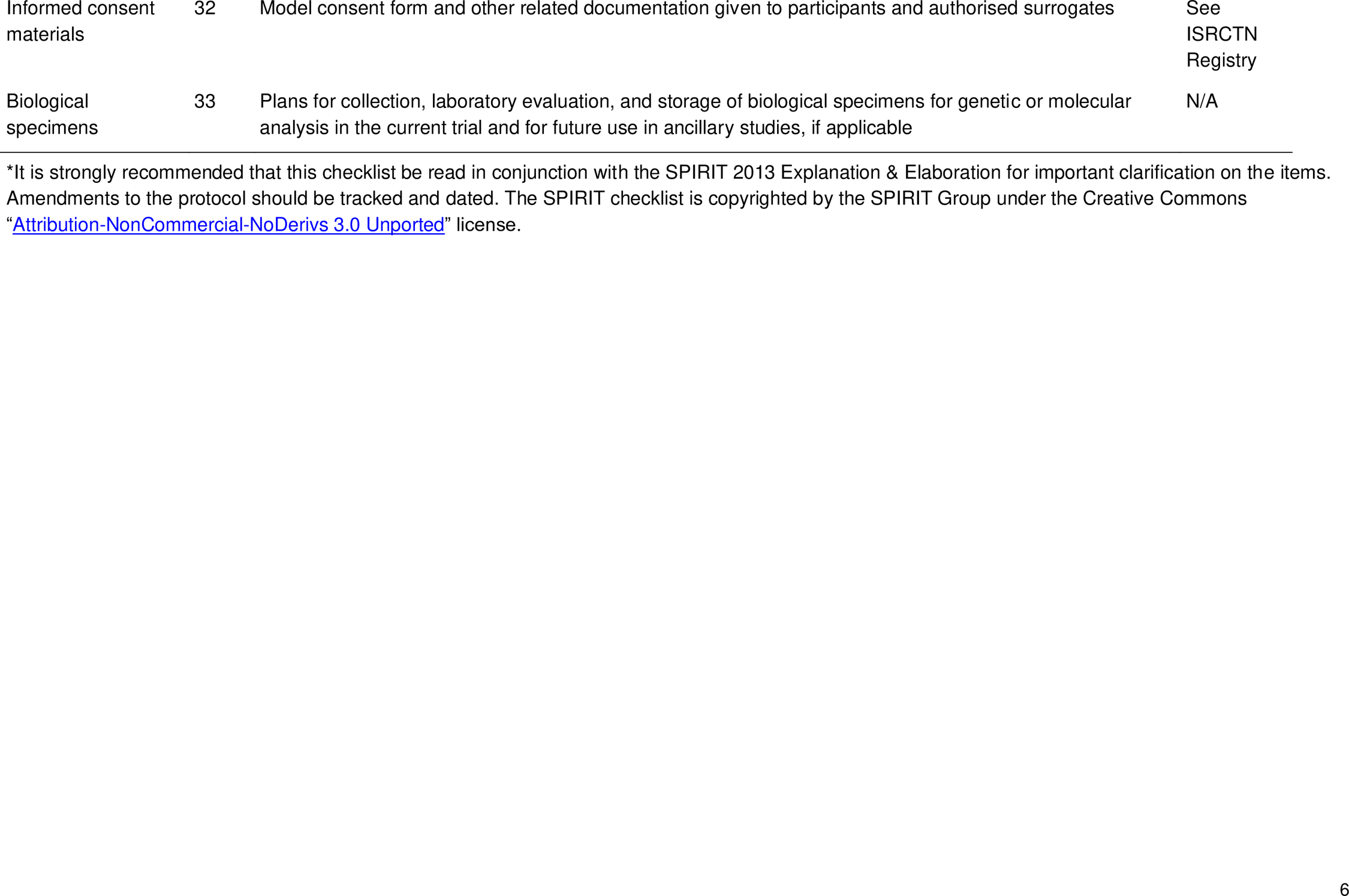

Supplement: S1 File — (TIF) [file pone.0275738.s001.tif]

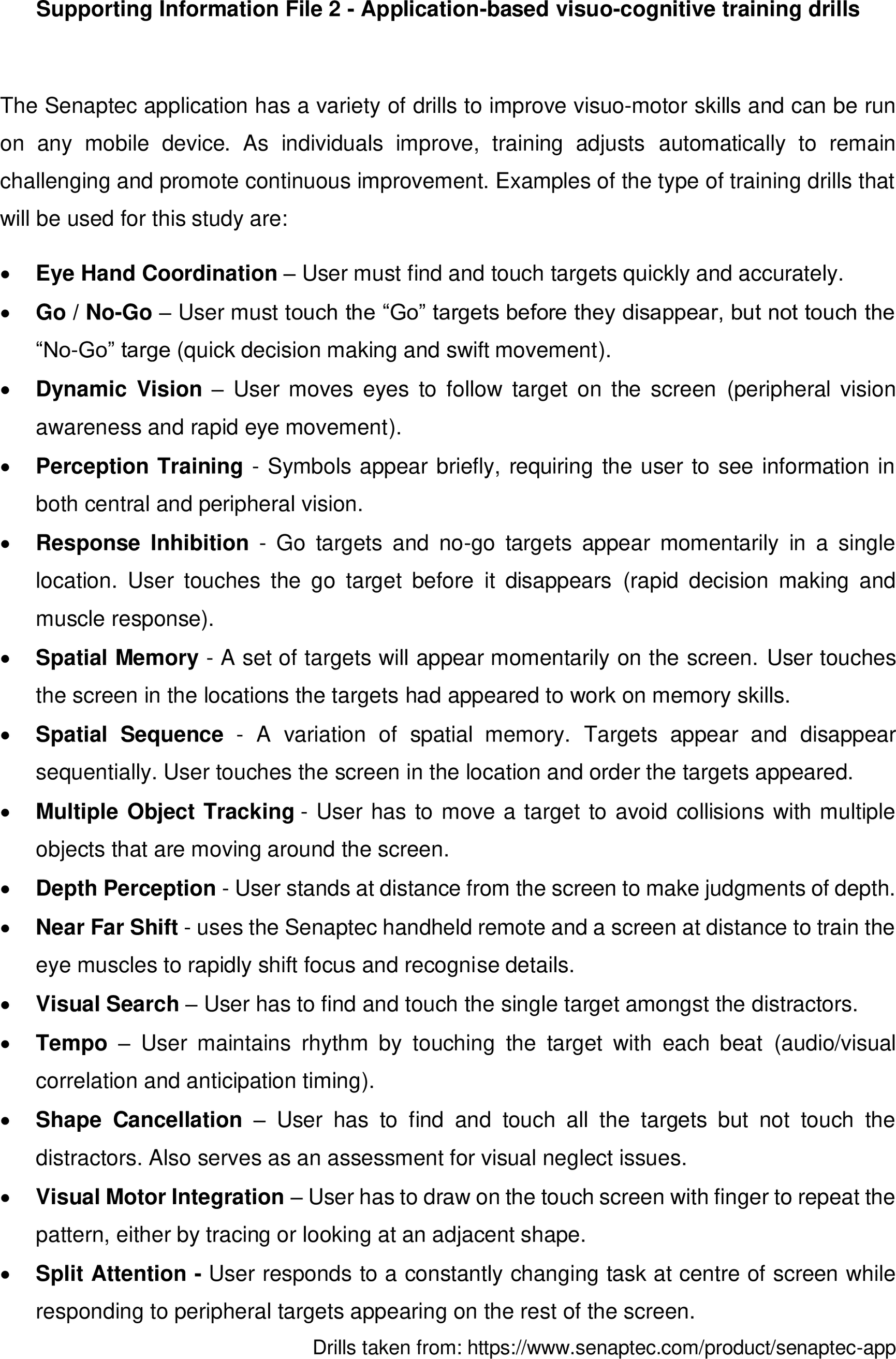

Supplement: S2 File — (TIF) [file pone.0275738.s002.tif]

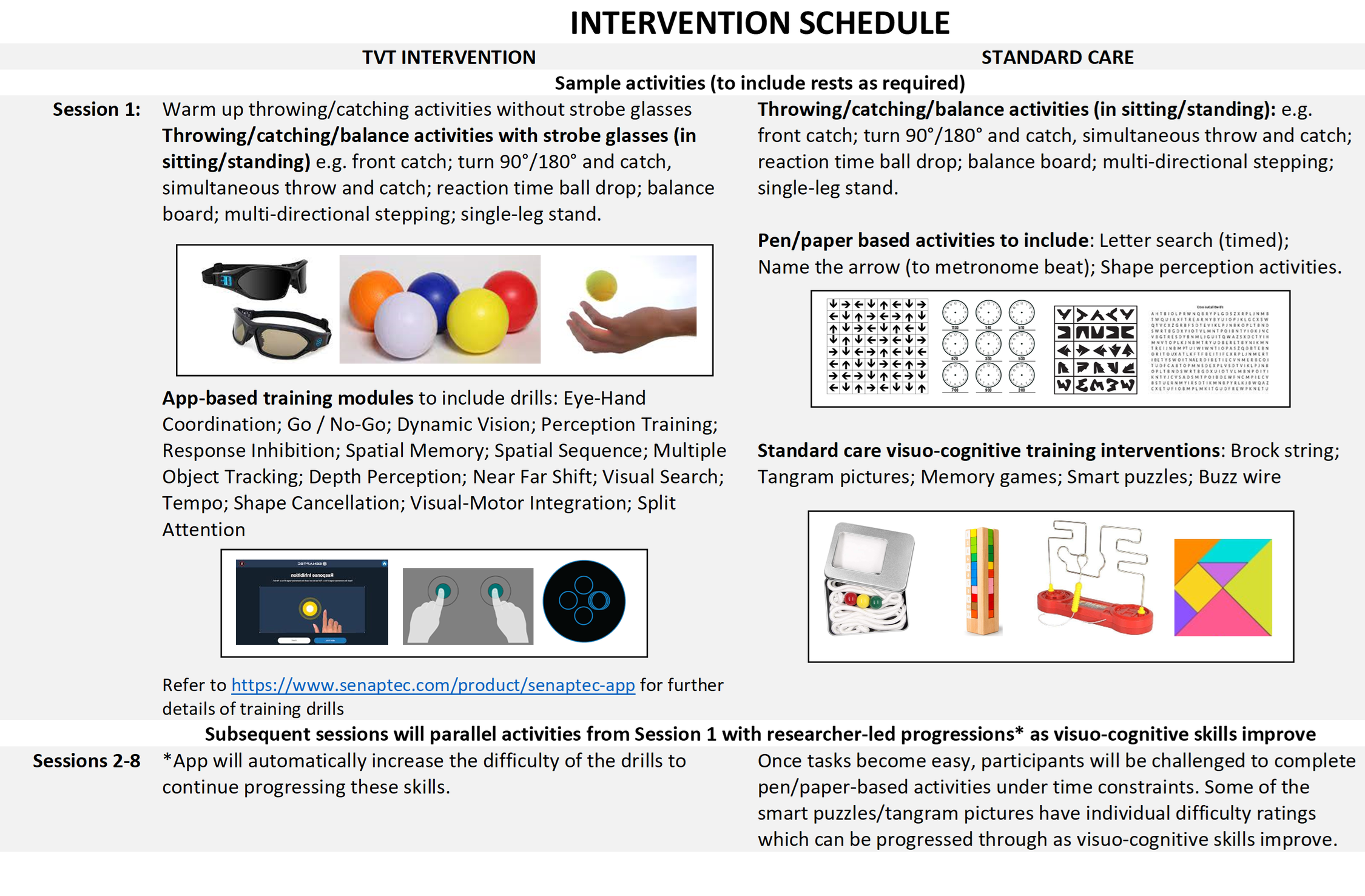

Supplement: S3 File — (TIF) [file pone.0275738.s003.tif]

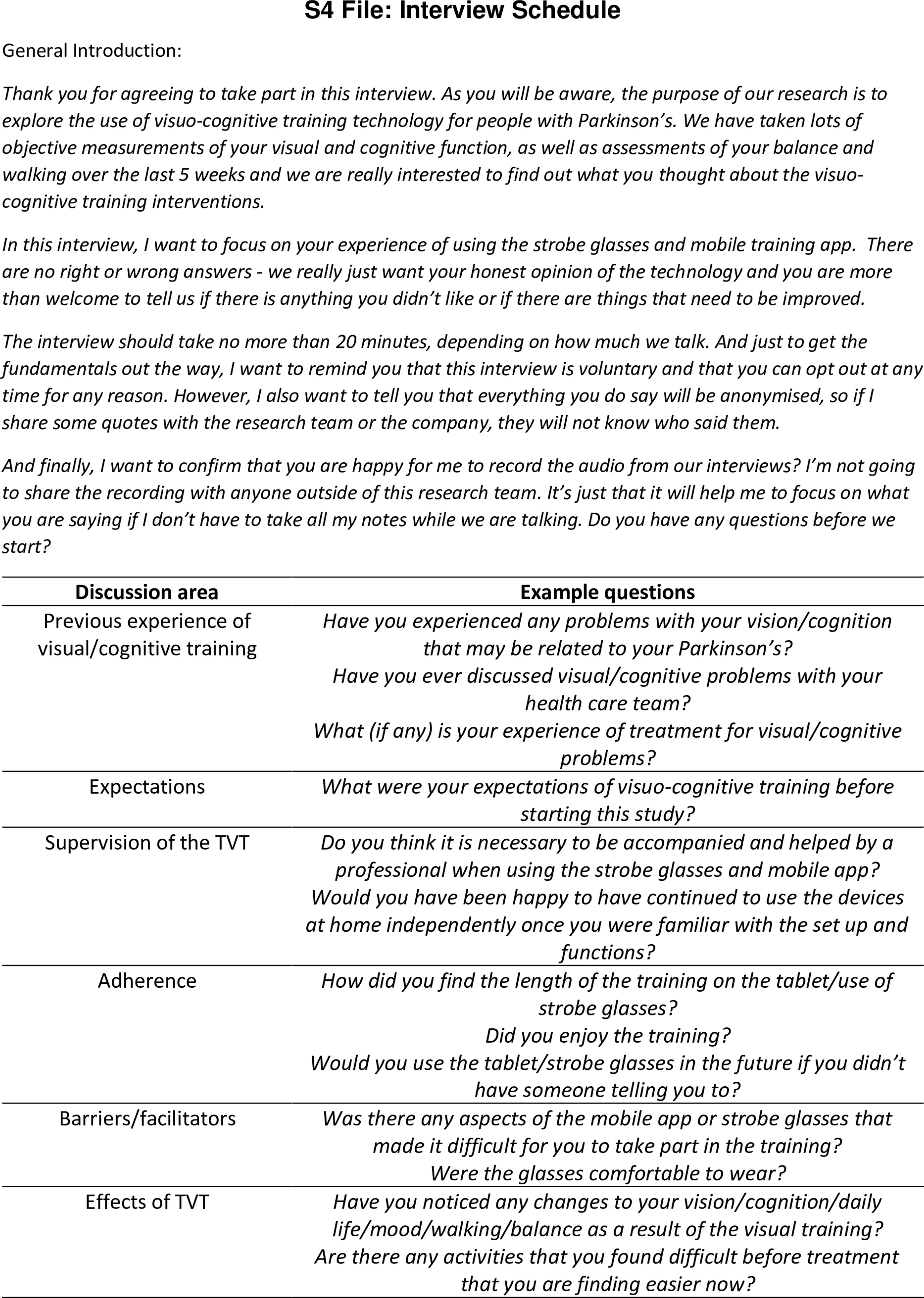

Supplement: S4 File — (TIF) [file pone.0275738.s004.tif]
