# Supplementary material for: Exploring the feasibility of technological visuo-cognitive training in Parkinson’s: Study protocol for a pilot randomised controlled trial
Source: PLoS One. 2022 Oct 7;17(10):e0275738. doi: 10.1371/journal.pone.0275738 (PMC9543984; doi:10.1371/journal.pone.0275738)
Supplement: S5 File — (DOCX) [file pone.0275738.s005.docx]

**Technological visual training in Parkinson's disease:**

**A pilot randomised trial.**

**Project Protocol Version 3.0**

**October 2021**

**INVESTIGATORS**

Dr Sam Stuart, PhD

Vice Chancellors Senior Research Fellow and Honorary Clinical Physiotherapist

Department of Sport, Exercise and Rehabilitation, Northumbria University, Northumberland Building (NB318), Newcastle upon Tyne, NE1 8ST

🕿 0191 227 3343

e-mail: sam.stuart@northumbria.ac.uk

Dr Rosie Morris, PhD

Senior Lecturer in Neurological Physiotherapy and Honorary Clinical Physiotherapist

Department of Sport, Exercise and Rehabilitation, Northumbria University, Northumberland Building (NB318), Newcastle upon Tyne, NE1 8ST

🕿 0191 237 3343

e-mail: [rosie.e.morris@northumbria.ac.uk](mailto:rosie.e.morris@northumbria.ac.uk)

Dr Gill Barry, PhD

Senior Lecturer

Department of Sport, Exercise and Rehabilitation, Northumbria University, Northumberland Building (NB318), Newcastle upon Tyne, NE1 8ST

🕿 0191 237 3343

e-mail: gill.barry@northumbria.ac.uk

Professor Richard Walker, MD

Consultant and Honorary Professor of Ageing and International Health

Northumbria Healthcare NHS foundation trust, North Tyneside General Hospital, Rake Lane, North Tyneside, NE29 8NH

🕿 0191 293 2709

e-mail: [Richard.Walker@northumbria-healthcare.nhs.uk](mailto:Richard.Walker@northumbria-healthcare.nhs.uk)

Julia Das, BSc (Hons)

PhD Candidate, Associate Lecturer in Physiotherapy and Honorary Clinical Physiotherapist

Department of Sport, Exercise and Rehabilitation, Northumbria University, Northumberland Building (NB318), Newcastle upon Tyne, NE1 8ST

🕿 0191 237 3343

e-mail: [julia.das@northumbria.ac.uk](mailto:julia.das@northumbria.ac.uk)

Dr Rodrigo Vitorio,PhD

Research Fellow

Department of Sport, Exercise and Rehabilitation, Northumbria University, Northumberland Building (NB318), Newcastle upon Tyne, NE1 8ST

🕿 0191 227 3343

e-mail: [rodrigo.vitorio@northumbria.ac.uk](mailto:rodrigo.vitorio@northumbria.ac.uk)

**Synopsis of Study**

| Study Title | **Technological visual training in Parkinson's disease: A pilot randomised parallel group trial.** |
| --- | --- |
| Internal ref. no. (or short title) | Visual training in Parkinson’s. |
| IRAS Number | 287526 |
| Study Design | A 4-week, single-site, parallel-group,RCT pilot involving human participants with a 1:1 allocation to either technological visual training or standard care group. |
| Study Participants | 40 people with Parkinson’s disease   - Hoehn & Yahr stage I-III |
| Planned Size of Sample (if applicable) | 40 |
| Follow up duration (if applicable) | N/A |
| Planned Study Period | May 2021-June 2023 |
| Research Question/Aim(s) | Aim I: Assess the feasibility of a technological visual training (TVT) intervention that requires wearing stroboscopic glasses and use of a tablet application in Parkinson’s.  Aim II: Measure the potential effects of TVT on visual function in Parkinson’s to determine if it produces greater benefit than a standard care intervention and assess the feasibility of a future large full-scale study. |

# FUNDING AND SUPPORT IN KIND

| **FUNDER** | **FINANCIAL AND NON FINANCIALSUPPORT GIVEN** |
| --- | --- |
| **Northumbria University**  **Senaptec, Beaverton, OR, USA** | **PhD studentship**  **£36,000 (£12,000 per year for PhD Studentship).**  **In kind support equipment provided.** |

**INTRODUCTION**

**LAY SUMMARY**

Parkinson’s disease is a common disorder that affects the brain. It can cause both movement and non-movement symptoms. Impairments of the eyes and visual system are a common non-movement symptom that affect some people with Parkinson’s (PwP). Visual disturbances can interfere with reading and driving, they can impact on walking and balance, and may contribute to hallucinations.

Research has shown PwP rely heavily on their vision for day to day activities. Visual disorders combined with balance problems can increase the risk of falls, leading to decreased physical activity and reduced quality of life for PwP.

Up to 80% of PwP experience some form of visual disturbance and yet very little research has been done around the assessment and treatment of visual disorders in Parkinson’s. However, there is now evidence that visual training technologies used in sporting contexts may be of benefit for PwP.

Stroboscopic visual training (SVT) is a form of training technology which is designed to improve the connections between an individual’s eyes, brain, and body. Individuals wear specialised glasses with lenses that flicker between clear and opaque. Studies in athletes have shown that SVT combined with mobile training applications can improve skills such as reaction times and hand-eye co-ordination.

We would like to find out what effect visual training has on vision in Parkinson’s. We will use both traditional visual training methods and state-of-the-art technology to assess and treat the visual system. We suggest that if visual training can improve aspects of eye function in PwP, it may also have the potential to impact on balance and walking.

This will be the first study of its kind to investigate the effects of SVT eyewear and visual training technology in a Parkinson’s population. Understanding the effects of visual training in PwP will help clinicians and therapists improve their treatment interventions and address a symptom of Parkinson’s that has historically received very little attention both from researchers and within clinical practice.

**SCIENTIFIC BACKGROUND**

**Parkinson’s disease leads to visual impairment**

Visual impairment is common in Parkinson’s disease, with over 80% of people with the condition reporting at least one visual symptom^1^. A large population-based study of older adults by Hamedani & Willis (2020) found that visual dysfunction is signiﬁcantly more common in people with Parkinson’s (PwP) than in the general population^2^.

Visual impairments in Parkinson’s are caused by abnormalities in nearly all aspects of visual function, ranging from initial targeting of gaze through head orientation and eye movements to visual processing involving both retina and higher order brain regions^3-6^.

Currently, visual assessments in PwP are conducted with eye charts, expensive devices or clinical pen and paper tests, which can be time-consuming and often require clinical expertise for interpretation. Novel modern technologies have the capability to measure visual functions using a single device within a short period of time, but these have yet to be trialled in PwP. This study will combine standardised clinical tools with touch-screen visual assessment technology (Senaptec Sensory Station, see *Appendix 1*) to objectively measure visual function and visuo-motor control in PwP.

**Visual impairments impact gait and balance**

Visual impairments are frequently associated with postural instability and gait disorders in PwP, which increase the risk of falls and lead to loss of independence and a decline in quality of life^6-10^.

In addition, deficits in the ability to use proprioceptive feedback in PwP result in greater reliance on an impaired visual system to carry out motor tasks^11-16^. The combination of visual-perceptual deficits and increased visual dependence has a significant impact when accompanied by primary symptoms of Parkinson’s such as bradykinesia, rigidity, tremor and postural instability^1,16,17^. These impairments reduce a person’s ability to compensate and adapt to motor disturbances, which can lead to a decline in functional tasks like walking, rising from a chair, and bed mobility^18^.

**Visual training in Parkinson’s**

There is growing interest in the assessment and training of the visual system to facilitate visuo-motor skill within sporting contexts, however relatively few studies have explored the effects of visual training (VT) within neurological populations^19-25^.

Preliminary research by Zampieri et. al. (2008, 2009) suggests that eye movement exercises combined with balance training may improve gait and gaze control in PwP^26,27^. More recent work has addressed eye-movement training in relation to saccade behaviour and its potential impact on motor-related Parkinson’s symptoms^28^. Despite increasing calls for further research into the area, treatment options for visual impairments in PwP remain limited to optimising dopaminergic therapy, traditional ophthalmological approaches and standard VT (e.g. eye movement training, hand-eye co-ordination, pen and paper perceptual tasks etc.)^4^.

**Technological visual training**

Recent years have seen an increase in the use of technology in neurorehabilitation^29^. In addition to mobile treatment apps, technological progression has led to the development of VT devices that could be deployed by clinicians or patients to improve visual function.

Stroboscopic VT (SVT) systems involve the intermittent reduction in visual input to create suboptimal visual conditions^21,30^, which can be used during standard visual training tasks or combined with mobile training applications^31^. The concept is based upon the premise that stroboscopic interruption of vision might enhance visual–motor control by reducing reliance on visual feedback and encouraging greater use of other senses^19,32^. The area has seen a substantial growth since 2011 as new and improved eyewear technology has been developed and used in sports vision training^25^.

Research performed on healthy adults, has demonstrated that SVT improves visual cognition^19^, anticipatory and reaction timing^21,33^, visual acuity^34^, hand-eye co-ordination^35^, visual attention and information encoding^20^. More recent research indicates that SVT may be more effective than conventional visuomotor training for improving visuomotor abilities in athletes^36^.

A recent study by Shalmoni & Kalron (2020) purports to be the first of its kind to investigate the use of SVT within a neurological population^37^. They examined the immediate effect of SVT on cognitive function, gait and static balance performance in people with multiple sclerosis (MS). Their findings demonstrated that SVT enhanced information processing speed immediately after training. While these findings were based on an MS population, the authors suggest that their research could have implications for the elderly and other neurological populations.

**Original contribution to knowledge**

Visual function is a major area of decline in Parkinson’s progression^38^. While there is emerging evidence to suggest VT may have a positive impact on visual function in clinical neurological populations^25^, there remains a significant gap in the literature around the effect of VT in PwP.

This pilot study aims to address this gap by exploring the potential role of VT in PwP. It will assess the feasibility and potential effectiveness of a technological visual training package involving the use of a mobile application and stroboscopic glasses (see *Appendix* 2) within a Parkinson’s population. To the authors’ knowledge, this will be the first study of its kind to investigate the use of technological visual training (TVT) in PwP.

Before performing a large RCT (phase III study), a pilot study needs to prove the feasibility and potential effectiveness of a technological visual training intervention involving the use of a mobile application and stroboscopic glasses within a Parkinson’s population^39^.

**Research aims**

The aims of this study are to

1. Assess the feasibility of a technological visual training (TVT) intervention that requires wearing stroboscopic glasses and use of a tablet application in PwP
2. Measure the potential effects of TVT on visual function in PwP to determine if it produces greater benefit than standard (non-technological) care and assess the feasibility of a future large full-scale study.

**Secondary Objectives**

1. To examine the impact of visual training on balance and gait (walking) outcomes in people with Parkinson's.
2. To explore other effects related to visual training including participants’ health status, quality of life, self-efficacy, levels of activity and participation.
3. To compare visual assessment outcomes using the Senaptec Sensory Station with standardised visual assessment tools currently used in clinical practice – a potential validation study.

**METHODS**

**TRIAL DESIGN**: A 4-week, single-site, parallel group, pilot RCT (allocation ration: 1:1)

**PARTICIPANTS**

A convenience sample of community-living ambulatory older adults with mild-to-moderate Parkinson’s disease (*n=40*) will be equally divided and randomly assigned (via random number generator) to either Group A (*n=20*) or Group B (*n=20*).

**Recruitment**

Potential participants will be recruited through Northumbria Healthcare NHS Foundations Trust (NHCFT) Parkinson’s service who care for over 1000 people with Parkinson’s disease and related conditions annually^40^. PwP will be identified through attendance at the Movement Disorders Clinics at Northumbria Healthcare NHS foundation trust, for which Professor Richard Walker (co-investigator of this study) is the clinical lead. Investigators will attend the North Tyneside Movement Disorders clinic weekly to work clinically as honorary clinical physiotherapists. If required, it will also be possible to recruit participants from other movement disorder clinics within NHCFT (such as Blyth, Morpeth and Wansbeck), where participants live sufficiently close to Northumbria University Coach Lane campus. Research personnel will be available at clinics as required to invite participants to consider the study. Additionally, we will recruit participants from Gateshead Health NHS trust, where PwP will be identified through the Movement Disorder Clinics at Gateshead, which are led by Dr Claire McDonald (Consultant Geriatrician). Verbal consent to their clinician will be required from patients in clinic for their details to be passed to the researchers. Research personnel will be available at clinics as required to invite participants to consider the study, and referrals will be made to the researchers during their attendance or via secure nhs.net email.If sufficiently interested, participants will be given a Participant Information Sheet (PIS) concerning the study. The invitation will be followed up by a telephone call during the week to assess willingness to participate. If willing, a mutually convenient time for assessment will be organised, and the invitation to attend will be extended to a carer or spouse.

If additional participants should be required, participants may also be recruited from previous Northumbria University studies where participants have consented to be contacted regarding participation in future studies, and from Parkinson’s UK. Parkinson’s UK will be provided with a lay summary of the study and a participant information sheet, with the contact details of the Principal investigator. Parkinson’s UK will place this study on the study recruitment section of their website and newsletters to advertise the study to potential participants. Potential participants who get in touch with the principal investigator to be involved in the study will be screened for inclusion and exclusion criteria over the telephone, and if eligible for the study, a mutually convenient time for assessment will be organised, and the invitation to attend will be extended to a carer or spouse.

The study has been accepted onto the NIHR portfolio, so the DeNDRoN network will also be involved for assistance with participant recruitment as required. Participants will be identified using the DeNDRoN Research Case Register. Registrants have a confirmed diagnosis of dementia or neurodegenerative disease (including Parkinson’s Disease) and have expressed their interest in hearing about local research relevant to their condition. Registrants have given their written or verbal consent for their medical notes to be accessed and screened by members of the DeNDRoN team to match them to a (NIHR portfolio) study. The register currently has several hundred PwP registered who have expressed their willingness to consider research participation, therefore it is anticipated the register will positively aid recruitment to this study if additional support is required.

Only members of the North East and N. Cumbria DeNDRoN team are permitted access to the register, therefore the initial approach will be made by them. If the patient expresses interest during the initial approach (consisting of a telephone call briefly outlining the study), with their consent, their contact details will be provided to the study team for further follow up.

The research team are confident that 40 participants can be recruited based on previous work by the Parkinson’s service which has recruited similar numbers to exercise trials for 12-week exercise interventions^41^. The study is expected to have a low attrition and a high compliance rate given that the intervention periods (4 weeks each arm) will be home-based with therapy supervision.

**Eligibility criteria for participants**

***Inclusion criteria:***

- Clinical diagnosis of Parkinson’s by a movement disorder specialist according to UK brain bank criteria (H&Y stage I-III^42^)
- Aged >50 years
- Able to walk and stand without support or assistance from another person
- Have adequate hearing/vision capabilities to allow participation in all aspects of study (if participant wears prescription glasses, they must be comfortable to remove these for short periods - up to 5 minutes at a time - in order to take part in activities whilst wearing strobe glasses)
- Stable medication for the previous 1 month and anticipated over a period of 6 months.

***Exclusion criteria:***

- History of epilepsy, seizures, migraines, severe motion sickness or sensitivity to light
- Psychiatric co-morbidity (e.g. major depressive disorder as determined by geriatric depression scale^43^– GDS-15: <10)
- Clinical diagnosis of dementia or other severe cognitive impairment
- History of stroke, traumatic brain injury, MS or neurological disorders other than Parkinson’s disease
- Acute lower back or lower extremity pain, peripheral neuropathy, rheumatic and orthopaedic diseases
- Unstable medical conditions including cardio-vascular instability in the past 6 months.
- If the participant is unable to comply with the testing protocol or currently participating in another interfering research project or undergoing any interfering therapy, they will not be recruited.
- Individuals who have *not* had opportunity to receive Covid-19 vaccine (please note, individuals may still be included in the study if they have been offered the vaccine but have declined due to personal circumstances).
- Anyone experiencing Covid-19 symptoms will be managed as per latest government guidelines.

**Informed consent**

The participant must personally sign and date the latest approved version of the Informed Consent form before any study specific procedures are performed.

Written versions of the Participant information sheet and Informed Consent form will be verbally discussed and presented to the participants detailing no less than: the exact nature of the study; what it will involve for the participant; the implications and constraints of the protocol; any risks involved in taking part. It will be clearly stated that the participant is free to withdraw from the study at any time for any reason without prejudice to future care, without affecting their legal rights, and with no obligation to give the reason for withdrawal.

The participant will then be given time to consider the information, and will have the opportunity to question the Investigator, their GP or other independent parties to decide whether they will participate in the study.

Written Informed Consent will then be obtained by means of participant dated signature and dated signature of the person who presented and obtained the Informed Consent. The person who obtained the consent must be suitably qualified and experienced and have been authorised to do so by the Principal Investigator. A copy of the signed Informed Consent form will be given to the participant. The original signed form will be retained at the study site.

**Settings and locations**

The setting for the study will be split between the clinical gait laboratory at Coach Lane Campus, Northumbria University (for assessment visits) and participant’s home address (for intervention sessions).

The Clinical Gait Laboratory is a dedicated facility for the investigation of gait, balance and mobility in healthy and clinical populations linking with the research themes of the Department of Sport, Exercise and Rehabilitation. The facility has all of the necessary equipment and space to allow this study to be conducted. Qualified physiotherapists will be present during the assessment visits. Reasonable travel expenses will be paid to participants for visits to the Gait Laboratory, with reimbursement based on the production of receipts, pre-paid taxi, or mileage allowances as appropriate.

**ASSESSMENTS**

A repeated measure design will be employed with assessments performed at the clinical gait lab on two separate sessions lasting between 2-3 hours. Participants will attend the lab for outcomes to be measured at the start (baseline) and at theend of the first 4 week intervention. Results will be compared between the groups.

1. Baseline assessment (up to 180 mins)

- Initial screening and standard clinical assessments
- Sensorimotor assessment using Senaptec Sensory Station (SSS)

1. Follow-up assessment (after 4 week intervention) (up to 180 mins)

- Standard clinical assessments
- Sensorimotor assessment using Senaptec Sensory Station (SSS)

**Initial Screening and Clinical Assessment Protocol**

*Past Medical History Interview*

The principal investigator or one of the investigators acting as his or her representative will provide the participants with the necessary oral and written explanations to obtain their signed/written informed consent before beginning the study.

Each participant will then be interviewed by a member of the study team. The interview will include questions regarding education level, falls history, activity level and side dominance as well as basic demographics such as age and gender.

Evaluation of cognition, visuomotor ability, disease severity, physical performance and quality of life will be performed by a physiotherapist from the research team with the following measures:

**Neuropsychological Tests**

***Geriatric depression scale (GDS-15)***

The short form GDS-15 which was created in 1986 by Sheikh and Yesavage will be used to evaluate subjects depression. This involves 15 questions about the mood of the subjects. Scores of 0 to 4 to be in the normal range, 5 to 9 to indicate mild depression, and 10 to 15 to indicate moderate to severe depression^44^.

***Montreal cognitive assessment (MoCA)***

Cognitive function will be assessed using standardized neuropsychological tests such as the MoCA; a rapid screening instrument for global cognitive dysfunction^45^. Different cognitive domains are assessed (attention and concentration, executive functions, memory, language, visuo-constructional skills, conceptual thinking, calculations, and orientation). The MOCA was found to be a valid instrument for cognitive screening in MCI and PD. In this study the MoCA will be used as a descriptive measure^45^.

***The Penn Parkinson's Daily Activities Questionnaire-15 (PDAQ-15)***

The PADQ-15 is a 15-item measure of cognitive instrumental activities of daily living for PwP derived from the original 50-item PDAQ^46^. It shows good discriminant validity across cognitive stages, correlates highly with global cognitive performance, and appears suitable to assess daily cognitive functioning in Parkinson’s disease^46^.

***Attention Computer Battery***

A computerised battery will be used to examine attention, via simple button pressing tasks that measure reaction time, such as simple reaction time, choice reaction time and digit vigilance. The attention battery is a valid means of testing attention and has been used in a number of studies involving both PD and cognitively impaired individuals^47^. The attention battery involves a series of computerised tests, which the subjects respond to on screen stimuli by pressing one of two buttons.

***Trail Making Test (TMT) Parts A and B***

The Trail Making Test is a visual attention assessment that has been used within various neurological and cognitively impaired groups since the 1940s. The test is done in two parts; A and B. The patient is asked to draw lines to consecutively numbered circles on one work sheet (Part A) and then to connect the same number of consecutively numbered and lettered circles on a separate work sheet, alternating between the two sequences (Part B). Subjects are told to work quickly and not to deviate from the appropriate sequence^48^. The examiners test the subjects and record the time, in seconds, needed to complete each part of the test. Any errors are counted and corrected by the examiner, with the timer still going during correction time.

***Benton’s Judgement of Line Orientation Test (JLO)***

The JLO is a high test-retest reliability test and has been shown to have good neuropsychological construct validity via neuroanatomical localization studies^49^[[1](#_ENREF_1)][[1](#_ENREF_1)][[1](#_ENREF_1)]. JLO is a test of visuospatial ability, which involves a subject viewing a set of numbered lines and then being shown two lines of the same orientation. They then have to name the numbers that the shown lines correspond to.

***Clock copying (e.g. Royall’s CLOX 1 and 2)***

Clock drawing (CLOX 1 and 2) test is a measure of cognitive impairment, which is an internally consistent measure that is easy to administer and has good reliability between raters. Clock drawing is seen as a visuospatial task linked with right parietal pathology. Participants are required to draw a clock with the numbers and arrows pointed at a particular time. Then the subjects have to copy a clock drawn by the researcher^50^.

**Visual Sensory Functions**

***Visual acuity (VA) (LogMAR)***

VA is measured binocularly used a standard LogMAR chart. Participants will be seated at a distance of 4m from the chart. Participants will be instructed to read aloud down the chart starting from the top left. All correct answers are recorded on a pre-set score sheet. Test is terminated if the participant makes 2 consecutive errors.

Final LogMAR calculated with the following formula: LogMAR = (score of the line before termination) – (0.02 x number of errors) + (0.02 x correct answers in the terminal line)

***Contrast sensitivity (CS) (Mars letter CS chart, Mars Percetrix™, New York, USA)***

CS will be measured using the Mars CS sheets placed on an adjustable holder. The sheet consists of 48 Latin letters of uniform height; the contrast from the white background decreases with subsequent letters. Room illumination is adjusted so that average CS sheet luminance is between 80 and 120cd/m² (measured via a luminance meter). Assessment is done binocularly with the average distance from the patients eyes being 50cm. Participants read aloud down the sheet starting at the top left. Errors are recorded on the pre-set score sheet and testing is terminated after 2 consecutive errors. Final logCS calculated using the following formula:

LogCS = (value of final correct letter before stopping) – (number of errors prior to stopping x 0.04).

***Senaptec Sensory Station (SSS), (Appendix 1)***

Visual and sensorimotor skills will be assessed on the Sensory Station which is a training station with a 50” screen. The SSS collects and analyses data relating to 10 sensory parameters (visual clarity/ contrast sensitivity/ depth perception/ near-far quickness/ perception span/ multiple object tracking/ reaction time/ target capture/ eye-hand coordination/ go-no-go).

**Disease Specific / Severity Tests**

***Hoehn & Yahr (H & Y)***

The Hoehn and Yahr rating scale is a widely used clinical rating scale, which defines broad categories of motor function in Parkinson’s disease^42^. All participants’ will be tested who are in H &Y stages I-III.

***The Unified Parkinson's Disease Rating Scale UPDRS-III (DOI: 10.1002/mds.22340)***

The Unified Parkinson's Disease Rating Scale (motor sub-score) part III, will be used to assess motor and non-motor features of PD and disease severity. The UPDRS is a short clinical assessment of disease severity / motor symptoms; higher scores reflect worsening disability.

***The new FOG questionnaire (new FOGQ) (DOI:https://doi.org/10.1016/S1353-8020(99)00062-0)***

Freezing of gait (FOG) will be evaluated using the new FOG questionnaire. This is a 10 item questionnaire intended to classify freezing of gait. The questionnaire has 3 parts; distinction of freezers from non-freezers, Freezing severity, frequency and duration and impact of freezing on daily life.

***Falls efficacy scale – International (FES-I)***

Fear of falling will be measured using the falls efficacy scale – international version. This is a short and valid measure of fear of falling in older adults, which assesses basic and demanding activities (both physical and social^51^. It consists of 16 scenarios (e.g. cleaning the house) and subjects must rate their fear of falling on a scale from 1 (Not at all concerned) to 4 (Very concerned).

***Parkinson’s Disease Questionnaire (PDQ-39)***

This 39 item questionnaire offers a patient reported measure of health status and quality of life^52^. It assesses how often people affected by Parkinson's experience difficulties across 8 dimensions of daily living including relationships, social situations and communication. It also assesses the impact of Parkinson's on specific dimensions of functioning and wellbeing.

**Physical/Functional Performance Tests**

***Mini Best Test***

This is a clinical balance test that has shown a high sensitivity in detecting balance impairments in elderly with Parkinson's disease^53^. It consists of 14 items, including tasks divided into four subcomponents: anticipatory postural adjustments, postural responses, sensory orientation, and dynamic gait. Items are scored from 0 (unable or requiring help) to 2 (normal) on an ordinal scale with the maximal total score of 28 points^53^.

***Gait and balance assessments***

Participants will stand still and walk in the gait laboratory while wearing several non-invasive wearable sensors that will monitor their balance and gait^54^. Rest breaks will be allowed as needed.  Participants will complete several standing and walking tasks in the laboratory, including (but not limited to) walking under single (usual walking) and dual task (speaking while walking) conditions^55^. A researcher will stand / walk close to participants in case of any balance loss.

***The Fatigue Severity Scale***

This is a nine-item questionnaire that assesses the eﬀect of fatigue on daily living. Each item is a statement on fatigue that the subject rates from 1, ‘completely disagree’ to 7, ‘completely agree’. It is brief, easy to administer, and demonstrates reliability and internal consistency in PwP^56^.

**Participation/Acceptability Measures**

***Pittsburgh Rehabilitation Participation Scale (PRPS)****^57^*

The PRPS measures participants’ participation in relation to the level of effort and motivation they demonstrate during a therapy session. This is an easy to administer 6-point scale whereby participants’ participation is scored from 1 (none) to 6 (excellent).

***Systems Usability Scale (SUS)****^58^*

This scale measures the usability of technology systems and devices by levels of agreement with ten statements that are scored using a five-point Likert scale from ‘strongly disagree’ to ‘strongly agree’.

**INTERVENTIONS**

Following screening and clinical assessment at the Gait Lab, participants will receive a telephone call from the researcher prior to starting the visits. This will allow participants to ask any further questions about the sessions following their assessments and to help them feel more familiar with the visiting therapist. It will also be an opportunity for the researcher to help the participant identify a suitable location in their home in which to undertake the subsequent training sessions in preparation for the first visit.

After the familiarisation phone call, the home visits will commence. Participants will be allocated to either Group A (technological visual training) or Group B (standard training). Both groups will receive two training sessions per week for four weeks in their home environment delivered by one of the researchers. Following this intervention, participants will be invited back to the Gait Lab for follow-up assessment. See *Figure 1* below for a summary for the study design.


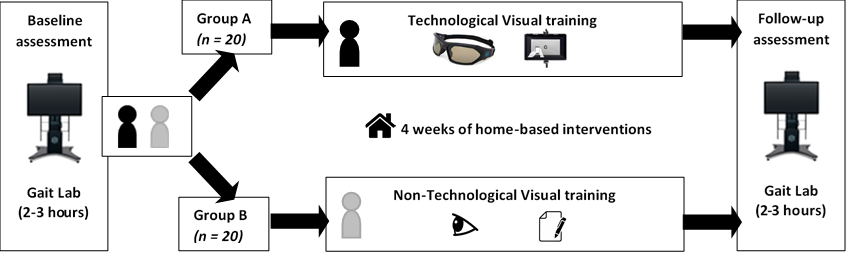


**Figure 1. Study design**

**Group A: Technological Visual Training (TVT)**

The experimental group will receive a total of 8 sessions of TVT over a 4 week period. Each session will last up to 60 min of which 20 mins will be dedicated to a series of visual training drills using a mobile tablet device (see *Appendix 3* for list of training drills), and a further 10 mins will be spent doing simple hand-eye co-ordination activities (e.g. throwing and catching) whilst wearing stroboscopic glasses. The remaining time will be factored in for rest periods as required. Activities will be supervised at all times by the researcher who will keep a running log of issues that may arise during the intervention period such as adverse events, changes in medication or any environmental factors that could affect participant engagement or performance.

**Group B: Standard (non technological) care intervention**

For the standard care group, participants will receive a total of 8 sessions of standard (non-technological) visual training over a 4 week period. As there is currently no accepted “gold standard” visual training approach for use in PwP, the control methodology for this study has been adapted from control conditions used in previous vision therapy trials and traditional therapy practice^31,59^. Participants will undergo 20 minutes of visual training drills involving a variety of visuo-motor and perceptual tasks (primary pen and paper), followed by a further 10 minutes of games requiring hand-eye coordination skills (e.g. throwing and catching). More details of the procedures can be found in *Appendix 4*.

**OUTCOMES**

For the purposes of this study, feasibility will be explored in relation to safety and acceptability of the TVT intervention. See *Appendix 5* for full description of feasibility outcomes for the study.

To estimate the potential effects of TVT in PwP, a set of clinical outcomes relevant to PwP will be assessed. The primary clinical outcome will be change in visual attention from baseline to follow-up assessed with the Trail Making Test and the Senaptec Sensory Station.

Secondary clinical outcomes will be changes in balance and gait, mobility, physical endurance, quality of life, cognition and falls-efficacy. Gait and balance characteristics will be recorded via wearable sensors (e.g. accelerometers) used during standing and walking under different conditions (including but not limited to single and dual tasks). Spatiotemporal gait characteristics (e.g., gait speed (m/s), variability (CV%), step/stride length (m), stride time (s), swing time (%), asymmetry, and step width (cm)) will be determined. Balance characteristics will include sway area (mm^2^), sway path length (mm) and sway velocity (mm/s).

Additional measures will be used to describe the characteristics of the participants in terms of disease status, multimorbidity and mood.

**SAMPLE SIZE**

The Consolidated Standards of Reporting Trials Group and bodies such as The National Institute for Health Research state that not all studies need a power-based sample size calculation but they do all need a sample size justification. As highlighted previously, this is a pilot study designed to test trial procedures and processes and to get estimates of parameters for a main trial sample size calculation. While we would hope to see trends in outcomes, the study is not designed to give a formal assessment of efficacy and there are few examples of visual training studies in PwP with which to base a sample size on. The majority of visual training and SVT studies have used relatively small cohorts (n<20), but have found statistically significant differences with intervention. Therefore, we propose recruiting a cohort of 40 participants (20 participants in each arm of the study) for the purposes of this study.

**Data Analysis**

The subjects or observations to be excluded, and the reason for their exclusion will be documented and approved by those responsible, prior to statistical analysis. Any exclusion documentation will be stored together with the remaining study documentation.

**Blinding**

Due to the pilot nature of this project and limited research personnel involved, it will not be possible to ensure blinding of investigators. Blinding will not be possible for participants, but they will be asked to refrain from mentioning the nature of the intervention they receive (technology versus standard) to the assessors in the Clinical Gait Laboratory.

**Statistical Analysis**

Statistical analysis will be undertaken using SPSS (v.26, IBM). All statistical tests will be carried out at the 5% two-sided level of significance. Demographic characteristics and baseline data will be summarized using descriptive statistics, including means, standard deviations, median, minimum, maximum and inter-quartile ranges for continuous or ordinal data and percentages for categorical data. The descriptive statistics will be tabulated and presented graphically for clarity.

The statistical methods to be used are outlined in the following paragraphs;

**Feasibility and Safety Analysis**

***Aim 1:***  ***Is a technological visual training intervention (using a combination of stroboscopic glasses and***  ***a tablet application) feasible, safe and acceptable for use in a Parkinson’s population?***

Data will be presented as mean +/- standard deviation (normally distributed data) or median (interquartile range). Differences between the standard care and TVT group will be compared by two-tailed t-test (e.g. mean duration of visual training undertaken at each one hour visit), or chi-square test (e.g. total number of differences in adverse events between arms, compliance/adherence).

Feasibility outcomes will also be reported both descriptively and narratively in relation to usability and acceptability of the TVT package (see *Appendix 5*).

**Efficacy Analysis**

***Aim 2: What are the potential effects of technological visual training in PwP and how do they compare to standard visual training techniques?***

The main focus of this aim of the study is to determine how the two treatments (TVT and standard care) influence participant outcomes over time, particularly visuomotor performance.

For this aim, data for the primary efficacy outcome (TMT) and secondary outcomes (balance, gait etc.) will be analysed using repeated measures analysis of covariance (ANCOVA), comparing the pre-intervention to post-intervention outcome. The dependent variable will be the outcome of interest (i.e., TMT), with age, gender, disease duration and H&Y stage as covariates. In case of any missing data, sensitivity analysis (last observation carried forward) will be used with delta-adjusting imputation.

**Safety Considerations**

All measurements and interventions are non-invasive and place the subject at no risk other than those that normally may occur during sitting, standing or walking. For some of the participants, particularly those who are not practicing any kind of physical exercise prior to the intervention, there is a slight possibility that they might feel some muscle soreness and fatigue after training. To prevent excessive fatigue, participants will be encouraged to take breaks as needed throughout all study procedures. They will also be advised to consider the potential impact of fatigue on their Parkinson’s symptoms when arranging their study visits.

It is possible that technological visual training may cause digital motion sickness (also known as *cyber sickness)*, a sensation similar to motion sickness which is caused by moving content on screens. Although this phenomenon is generally related to highly immersive technology (such as virtual reality (VR) headsets)^61^, the proposed study will deliver visual training at the most tolerable settings for individuals on the touch screen systems and strobe glasses to further minimise the risk of participants’ experiencing these symptoms. A recent study by Kim et. al. (2017) looking at the use of VR by older adults and PwP during walking showed that they were able to use immersive technology without experiencing adverse effects^62^ which provides reassurance that the non-immersive technological training component of the present study should not cause the participants any adverse effects.

The effects of using technology such as stroboscopic glasses as part of a visual training intervention in PwP is not yet known. Commonly recognised effects of prolonged exposure to strobe lighting such as that generated by flickering fluorescent lighting include headaches and eye fatigue, nausea and photosensitive epilepsy. Although participants will only be using the strobe glasses for up to 10 minutes at a time (including breaks), the eligibility criteria for this study has been designed to ensure that individuals who have a history of conditions that are known to be triggered by flashing/strobe lights (such as epilepsy or migraine) will be excluded from the study. Participants will also be given the opportunity to trial the strobe glasses prior to commencing procedures to ensure they can be tolerated. A qualified physiotherapist from the study team will always be with the participant whilst the technology is being used to monitor user-experience and ensure adequate rest periods are taken.

**Adverse Events:**

Any untoward medical occurrence, unintended disease or injury or any untoward clinical signs in participants whether or not related to the intervention will be recorded as an adverse event and managed according to the Health Research Authority (HRA) Guidance (see *Appendix 6* for a description of adverse events taken from HRA Guidance)**.**

Any serious adverse event (SAE) occurring to a research participant will be reported to the main Research Ethics Committee if the event is related to the administration of the research procedures and unexpected.

Recording of adverse events will start from the signature of the informed consent until the participant has completed or passed through the Day 7 ± 1 days follow-up window. Information and assessments relating to the adverse event will be recorded in the adverse event section of the Case Report Form as per HRA Guidance and will be monitored until resolution or stabilization.

In the unlikely event of any serious adverse event occurring during this study, the University will be informed immediately (and no later than 72 hours after first awareness of the research team) and a written report provided. The university (acting as the Local Sponsor) will follow local reporting guidelines.

**COVID-19:**

This study will follow the most up to date UK Government, NHS and Northumbria University Guidelines in all matters relating to Covid-19. This will include guidance on personal protective equipment (PPE) for both participants and researchers, and social distancing within the large gait lab space (which has an active ventilation system) and within participants’ own homes. The study involves the use of different wearable technologies, which can largely be applied by the participant themselves, without the researcher needing to be in close proximity. Only two researchers will be present during testing sessions in the Gait Lab to reduce the number of people in the room, and where possible carers or spouses who attend with subjects will be asked to wait outside in the seating area. All participants (and carers or spouses) will be asked if they have had any COVID-19 symptoms within the past several weeks over the phone the day before the visit, and this will be confirmed again on arrival at the Gait Lab. Researchers will wear appropriate PPE during the assessment, and the subjects and researchers will wash their hands upon touching others, or entering or leaving the room (alcohol gel will be available throughout the visit to be used when necessary). The gait laboratory will undergo a ‘deep clean’ for 30 minutes after the participant has attended the laboratory.

For interventions in the home environment, researchers will adhere to Government Guidelines and local NHS protocols. A full risk assessment will be undertaken prior to commencing any interventions in the home environment which will include any measures relating to Covid-19 restrictions that are in place at the time of formulating the assessment such as social distancing guidelines and infection control measures.

**Data Protection and Patient Confidentiality**

The study will comply with the General Data Protection Regulation (GDPR) and Data Protection Act 2018, which require data to be de-identified as soon as it is practical to do so. The processing of the personal data of participants will be minimised by making use of a unique participant study number only on all study documents and any electronic database(s). All data samples collected as part of this study will be anonymised with participants being assigned a unique study number (e.g. PD01, PD02 etc.). All electronically stored data (e.g. videos) will be named using the individuals study number to ensure confidentiality. The only information we will retain for our database will be the age and sex of participants and whether they are a patient. We will keep one hard copy of the assessment in locked filing cabinets in the Clinical Gait Laboratory, Coach Lane, Northumbria University. This is the only place where we store any personal details like names and addresses. This information is kept locked away and is only available to people directly running the study. These people will treat the information in the strictest confidence. Dr Samuel Stuart, the Principal investigator of this study, is ultimately responsible for the protection of this information.

The Principal investigator (Dr Stuart) will ensure that this study is conducted in accordance with relevant regulations and with Good Clinical Practice. The results of any tests will be kept strictly confidential. This data can only be accessed directly by the development team on the application and will be securely password protected. The data will be kept and stored according to the university's regulations and will be destroyed as such when the study is complete. There is no personal or identifiable data stored in the application itself, as that would be a breach of data security. Information is also kept in accordance to GDPR and will be destroyed according to the appropriate timescales. Once the study has completed its main objectives, data will be stored for 10 years after which it will be disposed of.

**Data Sharing**

The dataset may be used for secondary analysis with the consent of the participants (on consent form), and all participant documentation will reflect the potential future use of these data in research. To maximise impact from this research study, following the end of the study anonymous data will be securely stored on an external online repository, such as FigSHARE with which Northumbria University has an existing partnership. Secondary researchers will be required to reference the data being used.

**Access to Data**

Direct access will be granted to authorised representatives from the host institution for monitoring and/or audit of the study to ensure compliance with regulations. The anonymous dataset may be used for secondary analysis with the consent of the participants (via consent form). All patient documentation will reflect the future use of these data in research.

**Project Management**

The study will be run by Dr Samuel Stuart (Principal Investigator) and researchers within his team. The Northumbria University team will be responsible for ensuring progress of the study in relation to administrative, clinical and academic issues. All published output from the study will acknowledge researchers involved.

**Insurance Indemnity**

The University has a specialist insurance policy in place which would operate in the event of any participant suffering harm as a result of their involvement in the research (U.M. Association Limited)**.**

**Peer Review and Patient and Public Involvement**

This project and protocol design (including cohorts and statistical analysis) has been internally peer reviewed by the study investigators plus an independent expert from the University, with feedback incorporated into the study design and protocol.

Six people with Parkinson’s were contacted to provide Patient and Public Involvement (PPI) for this study. The participants were recruited through Parkinson’s UK and attended a virtual focus group to provide valuable feedback on the study concept, design and protocol. The participants were very enthusiastic about the aims of the study, particularly as it provides a novel intervention and addresses a non-motor symptom of Parkinson’s that is generally quite neglected in clinical practice. They did express some concerns about the wearability and acceptability of the strobe glasses, which we addressed by amending the eligibility criteria to include individuals who were able to participate in activities for short periods without prescriptive eyewear as well as changing the focus of the aims to include feasibility and safety outcomes.

Both the Plain English Summary and the Participant Information Sheet (PIS) were considered clear, readable and free of jargon, although it was acknowledged that several abbreviations were used throughout the text and the term *People with Parkinson’s* (PwP) was deemed more appropriate than *Parkinson’s disease* (PD). The documents have been revised accordingly and a short glossary has also been included at the end of the PIS which covers any technical terms and abbreviations.

The participants did not raise any issues regarding retention and adherence to the study because a therapist would be delivering the intervention sessions, but they did highlight that changes in medication/lifestyle behaviours may occur during two 6-week interventions which could potentially cause anomalies in the data collected. This has been addressed by reducing the proposed intervention phases to two 4-week periods and ensuring that the researcher keeps a concurrent log of any individual events that may impact results.

Participants were satisfied with the length of the individual visual training interventions providing adequate rest periods were included in the sessions and study participants were made aware of the potential for post training fatigue. A section in the PIS has been added to ensure that participants are aware of the potential impact on fatigue levels both during intervention sessions and in the days between visits.

### **Dissemination policy**

The data arising from the study is owned by Northumbria University:

On completion of the study, the data will be analysed and tabulated and a Final Study Report prepared.

The final study report can be accessed in the office of the PI, Dr Samuel Stuart.

- All participating investigators have rights to publish any of the study data, with agreement from the other investigators.
- The participants will be notified of the outcome of the study via a specifically designed newsletter.
- Participants can specifically request results which will be provided after the Final Study Report had been compiled.
- The study protocol, full study report, anonymised participant level dataset, and statistical code for generating the results will be made available on request.

**Authorship eligibility guidelines and any intended use of professional writers**

To warrant authorship on publications all study investigators will be examined for the following four authorship criteria:

- Substantial contributions to the conception or design of the work; or the acquisition, analysis, or interpretation of data for the work; AND
- Drafting the work or revising it critically for important intellectual content; AND
- Final approval of the version to be published; AND
- Agreement to be accountable for all aspects of the work in ensuring that questions related to the accuracy or integrity of any part of the work are appropriately investigated and resolved.

**REFERENCES**

1. Borm, C., Visser, F., Werkmann, M., de Graaf, D., Putz, D., Seppi, K., Poewe, W., Vlaar, A., Hoyng, C., Bloem, B. R., Theelen, T., & de Vries, N. M. Seeing ophthalmologic problems in Parkinson disease: Results of a visual impairment questionnaire. *Neurology*, 2020, 94(14): 1539–1547.
2. Hamedani, A.G. & Willis, A.W. Self-reported visual dysfunction in Parkinson disease: the Survey of Health, Ageing and Retirement in Europe*. European Journal of Neurology,* 2020, 27(3): 484-489.
3. Archibald, N.K., Clarke, M.P., Mosimann, U.P., Burn, D.J. The retina in Parkinson's disease. *Brain,* 2009, 132: 1128–1145.
4. Savitt, J. & Mathews, M. Treatment of Visual Disorders in Parkinson Disease*. Current Treatment Options in Neurol*ogy, 2019, 20(8): 30.
5. Stuart, S., Lord, S., Hill, E., Rochester, L. Gait in Parkinson’s disease: a visuo-cognitive challenge, *Neuroscience and Biobehavioural Reviews*, 2016, 62, 76-88.
6. Weil, R.S., Schrag, A.E., Warren, J.D., Crutch, S.J., Lees, A.J., & Morris, H.R. Visual dysfunction in Parkinson’s disease. *Brain*, 2016, 139(11): 2827-2843.
7. Ellis, T., de Goede, C.J., Feldman, R.G., Wolters, E.C., Kwakkel, G., Wagenaar, R.C. Efficacy of a physical therapy program in patients with Parkinson’s disease: A randomized controlled trial. *Archives of Physical Medicine and Rehabilitation,* 2005, 86(4): 626-632.
8. Hamedani, A. G., Abraham, D. S., Maguire, M. G., & Willis, A. W. Visual Impairment Is More Common in Parkinson’s Disease and Is a Risk Factor for Poor Health Outcomes. *Movement Disorders*, 2020, 35(9): 1542-1549.
9. Rumalla, K., Gondi, K.T., Reddy, A.Y., Mittal, M.K. Association of Parkinson’s disease with hospitalization for traumatic brain injury. *International Journal of Neurosciences*, 2017, 127(4): 326–33.
10. Wood, B.H., Bilclough, J.A., Bowron, A., Walker, R.W. Incidence and prediction of falls in Parkinson’s disease: a prospective multidisciplinary study. *Journal of Neurology, Neurosurgery & Psychiatry*, 2002, 72(6): 721–5.
11. Abbruzzese, G. & Berardelli, A. Sensorimotor integration in movement disorders. *Movement Disorders,* 2003, 18(3): 231-240.
12. Almeida, Q.J., Frank, J.S., Roy, E.A., Jenkins, M.E., Spaulding, S., Patla, A.E., Joget, M.S. An evaluation of sensorimotor integration during locomotion toward a target in Parkinson’s disease. *Neuroscience*, 2005, 134: 283–293.
13. Azulay, J.P., Mesure, S., Amblard, B., Pouget, J. Increased Visual Dependence in Parkinson’s Disease. *Perceptual and Motor Skills*, 2002, 95(3_suppl): 1106-1114.
14. Davidsdottir, S., Cronin-Golomb, A., Lee, A. Visual and spatial symptoms in Parkinson's disease. *Vision Research,* 2005, 45(10): 1285-96.
15. Halperin, O., Israeli-Korn, S., Yakubovich, S., Hassin-Baer, S., Zaidel, A. Self-motion perception in Parkinson's disease. *European Journal of Neuroscience,* 2020, 00: 1–12.
16. Jacobs, J.V., Horak, F.B. Abnormal proprioceptive-motor integration contributes to hypometric postural responses of subjects with Parkinson’s disease. *Neuroscience*, 2006, 141: 999–1009.
17. Gelb, D.J., Oliver, E., Gilman, S. Diagnostic Criteria for Parkinson Disease. *Archives of Neurology*, 1999, 56(1): 33–39.
18. Alenicova, O.A., Likhachev, S.A., Davidova, O.I. Clinical Significance and Pathogenesis of Visual Impairment in Parkinson's Disease. *Journal of Neurology & Stroke*, 2017, 7(7): 00266.
19. Appelbaum, L. G., Schroeder, J. E., Cain, M. S., & Mitroff, S. R. Improved Visual Cognition through Stroboscopic Training. *Frontiers in Psychology*, 2011, 2: 276.
20. Appelbaum, L.G., Cain, M.S., Schroeder, J.E., Darling, E.F., Mitroff, S.R. Stroboscopic visual training improves information encoding in short-term memory. *Attention, Perception, & Psychophysics*, 2012, 74: 1681–1691.
21. Smith, T.Q. & Mitroff, S.R. Stroboscopic training enhances anticipatory timing. *International Journal of Exercise Science,* 2012, 5(4): 344–353.
22. Appelbaum, L.G. & Erickson, G. Sports vision training: A review of the state-of-the-art in digital training techniques. *International Review of Sport and Exercise Psychology*, 2018, 11(1): 160-189.
23. Mitroff, S.R., Friesen, P., Bennett, D., Yoo, H., Reichow, A.W. Enhancing ice hockey skills through stroboscopic visual training: a pilot study. *Athletic Training & Sports Health Care,* 2013, 5: 261–264.
24. Wang, L., Krasich, K., Bel-Bahar, T., Hughes, L., Mitroff, S. R., & Appelbaum, L. G. Mapping the structure of perceptual and visual-motor abilities in healthy young adults. *Acta Psychologica*, 2015, 157: 74–84.
25. Wilkins, L. & Appelbaum, L.G. An early review of stroboscopic visual training: insights, challenges and accomplishments to guide future studies, *International Review of Sport and Exercise Psychology*, 2020, 13:1: 65-80
26. Zampieri, C. & Di Fabio, R.P. Balance and eye movement training to improve gait in people with progressive supranuclear palsy: quasi randomized clinical trial. *Physical Therapy,* 2008, 88: 1460–1473.
27. Zampieri, C. & Di Fabio, R.P. Improvement of gaze control after balance and eye movement training in patients with progressive supranuclear palsy: a quasi-randomized controlled trial. *Archives of Physical Medicine & Rehabilitation,* 2009, 90: 263-70.
28. Camacho, P. B., Carbonari, R., Shen, S., Zadikoff, C., Kramer, A. F., & López-Ortiz, C. Voluntary Saccade Training Protocol in Persons With Parkinson’s Disease and Healthy Adults. *Frontiers in Aging Neuroscience*, 2019, 11: 77.
29. M. Linares-del Rey, L. Vela-Desojo, R. Cano-de la Cuerda. Mobile applications in Parkinson's disease: a systematic review. *Neurology*, 2019, 34(1): 38-54.
30. Bennett, S. J., Hayes, S. J., & Uji, M. Stroboscopic Vision When Interacting With Multiple Moving Objects: Perturbation Is Not the Same as Elimination. *Frontiers in Psychology*, 2018, 9: 1290.
31. Liu, S., Ferris, L.M., Hilbig, S., Asamoa, E., LaRue, J.L., Lyon, D., Connolly, K., Port, N., Appelbaum, L.G. Dynamic vision training transfers positively to batting performance among collegiate baseball batters. *Psychology of Sport and Exercise*, 2020, 51.
32. Ellison, P., Jones, C., Sparks, A. Murphy, P.N., Page, R.M., Carnegie, E., Marchan, D.C. The effect of stroboscopic visual training on eye–hand coordination. *Sports Sciences for Health*, 2020, 16: 401–410.
33. Wilkins, L., Nelson, C. & Tweddle, S. Stroboscopic Visual Training: a Pilot Study with Three Elite Youth Football Goalkeepers. *Journal of Cognitive Enhancement*, 2017, 2: 3-11.
34. Holliday, J. (2013). Effect of stroboscopic vision training on dynamic visual acuity scores: Nike Vapor Strobe eyewear. All Graduate Plan B and other Reports, paper 262.
35. Jones, C., Carnegie, E., & Ellison, P. (Accepted/In press). The Effect of Stroboscopic Vision Training on Eye-Hand Coordination. Poster session presented at British Psychological Society (BPS) Division of Sport & Exercise Science Conference, Cardiff, United Kingdom. <http://www.bps.org.uk/events/conferences/division-sport-and-exercise-psychology-conference-0>, 2016.
36. Hülsdünker, T., Rentz, C., Ruhnow, D., Käsbauer, H., Strüder, H.K., Mierau, A. The Effect of 4-Week Stroboscopic Training on Visual Function and Sport-Specific Visuomotor Performance in Top-Level Badminton Players. *International Journal of Sports Physiology and Performance,* 2019, 14(3): 343-350.
37. Shalmoni, N. & Kalron, A. The immediate effect of stroboscopic visual training on information-processing time in people with multiple sclerosis: an exploratory study. *Journal of Neural Transmission*, 2020, 127: 1125–1131.
38. Terao, Y., Fukuda, H., Yugeta, A., Hikosaka, O., Nomura, Y., Segawa, M., Hanajima, R., Tsuji, S., Ugawaet, Y. Initiation and inhibitory control of saccades with the progression of Parkinson’s disease – Changes in three major drives converging on the superior colliculus. *Neuropsychologia*, 2011, 49: 1794–1806.
39. Thabane, L., Ma, J., Chu, R, Cheng J, Ismaila A, Rios LP, et al. A tutorial on pilot studies: the what, why and how. *BMC Med Res Methodol*. 2010; 10:1.
40. Hand A, Oates LL, Gray WK, Walker RW. Understanding the Care Needs and Profile of People Living at Home With Moderate to Advanced Stage Parkinson Disease. *J Geriatr Psychiatry Neurol*. 2018, 31(5): 237-247.
41. O'Callaghan A, Harvey M, Houghton D, Gray WK, Weston KL, Oates LL, Romano B, Walker RW. Comparing the influence of exercise intensity on brain-derived neurotrophic factor serum levels in people with Parkinson's disease: a pilot study. *Aging Clin Exp Res*. 2020, 32(9): 1731-1738.
42. Goetz, C.G., Poewe, W., Rascol, O., Sampaio, C., Stebbins, G.T., Counsell, C., Giladi, N., Holloway, R.G., Moore, C.G., Wenning, G.K., Yahr, M.D., Seidl, L. "Movement Disorder Society Task Force Report on the Hoehn and Yahr Staging Scale: Status and Recommendations. The Movement Disorder Society Task Force on Rating Scales for Parkinson's Disease". *Movement Disorders*, 2004, 19(9): 1020–1028.
43. Weintraub, D., Oehlberg, K. A., Katz, I. R., & Stern, M. B. Test Characteristics of the 15-Item Geriatric Depression Scale and Hamilton Depression Rating Scale in Parkinson Disease. *The American Journal of Geriatric Psychiatry*, 2006, 14(2): 169–175.
44. Aikman, G.G. & Oehlert, M.E. Geriatric Depression Scale, *Clinical Gerontologist*, 2001, 22: 3-4, 63-70.
45. Dalrymple-Alford, J.C., et al. The MoCA: Well-suited screen for cognitive impairment in Parkinson disease. *Neurology*, 2010, 75: 1717-1725.
46. Brennan, A.L., Siderowf, J.D. Rubright, J. Rick, N. Dahodwala, J.E. Duda, H. Hurtig, M. Stern, S.X. Xie, L. Rennert, J. Karlawish, J.A. Shea, J.Q. Trojanowski, D. Weintraub, Development and initial testing of The Penn Parkinson's Daily Activities Questionnaire, *Mov. Disord*. 2016, 31 (1) 126e134.
47. Wesnes, K.A., McKeith, I., Edgar, C., Emre, M., Lane, R. Benefits of rivastigmine on attention in dementia associated with Parkinson disease. *Neurology*, 2005. 65: 1654–1656.
48. Fals-Stewart, W. An interrater reliability study of the Trail Making Test (Parts A and B). *Perceptual and Motor Skills*, 1992, 74(1): 39-42.
49. Calamia, M., et al., *Developing a short form of Benton's Judgment of Line Orientation Test: an item response theory approach.* *Clin Neuropsychol*, 2011, 25(4): 670-84.
50. Royall, D.R., J.A. Cordes, and M. Polk, *CLOX: an executive clock drawing task.* *J Neurol Neurosurg Psychiatry*, 1998, 64: 588-594.
51. Yardley, L., et al., *Development and initial validation of the Falls Efficacy Scale-International (FES-I).* *Age Ageing*, 2005, 34(6): 614-9.
52. Peto, V., Jenkinson, C. & Fitzpatrick, R. PDQ-39: a review of the development, validation and application of a Parkinson’s disease quality of life questionnaire and its associated measures. *J Neurol,* 1998. 245**:**S10–S14.
53. Leddy, A.L., Crowner, B.E., Earhart, G.M. Utility of the Mini-BESTest, BESTest, and BESTest sections for balance assessments in individuals with Parkinson disease. *Journal of Neurologic Physical Therapy, 2011,* 35(2): 90-7.
54. Rosenfeldt A.B., Penko, A.L., Bazyk, A.S., Streicher, M.C., Dey, T., Alberts, J.L. The Two Minute Walk Test Overground and on a Self-Paced Treadmill Detects Dual Task Deficits in Individuals With Parkinson's Disease. *J Aging Phys Act*., 2019, 1;27(4): 843-847.
55. Light, K.E., Bebrman, A.L., Thigpen, M., & Triggs, W.J. The 2-minute walk test: a tool for evaluating walking endurance in clients with Parkinson's disease. *Journal of Neurologic Physical Therapy*, 1997, 21(4), 136.
56. Friedman, J.H., Alves, G., Hagell, P., Marinus, J., Marsh, L., Martinez-Martin, P., Goetz, C.G., Poewe, W., Rascol, O., Sampaio, C., Stebbins, G., Schrag, A. Fatigue rating scales critique and recommendations by the Movement Disorders Society task force on rating scales for Parkinson's disease. *Mov Disord*. 2010, 15; 25(7): 805-22.
57. Lenze, E.J., Munin, M.C., Quear, T., Dew, M.A., Rogers, J.C., Begley, A.E., Reynolds, C.F. The Pittsburgh Rehabilitation Participation Scale: reliability and validity of participation in acute rehabiltaion. Archives of Physical Medicine & Rehabilitation, 2004, 85(3): 380-384.
58. Brooke, J. SUS- A quick and dirty usability scale. Usability Evaluation in Industry. 1996, 189: 4-7.
59. Scheiman M, Mitchell GL, Cotter S, et al. The convergence insufficiency treatment trial: Design, methods, and baseline data. *Ophthalmic Epidemiol*. 2008;15(1):24-36.
60. King, L. A., Mancini, M., Smulders, K., Harker, G., Lapidus, J. A., Ramsey, K., Carlson-Kuhta, P., Fling, B. W., Nutt, J. G., Peterson, D. S., & Horak, F. B. Cognitively Challenging Agility Boot Camp Program for Freezing of Gait in Parkinson Disease. *Neurorehabilitation and neural repair*, 2020, *34*(5), 417–427.
61. Sharples, S., Cobb, S., Moody, A., Wilson, J.R. Virtual reality induced symptoms and effects (VRISE): Comparison of head mounted display (HMD), desktop and projection display systems. *Displays*, 2008; 29: 58–69.
62. Kim, A., Darakjian, N. & Finley, J.M. Walking in fully immersive virtual environments: an evaluation of potential adverse effects in older adults and individuals with Parkinson’s disease. *J Neuro Engineering Rehabil, 2017,* 14: 16.

**List of Appendices**

| *Appendix 1* | **Senaptec Sensory Station** |
| --- | --- |
| *Appendix 2* | **Stroboscopic Glasses and Mobile Training App** |
| *Appendix 3* | **App-based Visual Training Drills** |
| *Appendix 4* | **Standard care Interventions** |
| *Appendix 5* | **Feasibility Outcomes** |
| *Appendix 6* | **Safety Reporting Procedures for Adverse Events** |

**Appendix 1 – Senaptec Sensory Station**

The Sensory Station is an evaluation & training station which assesses 10 visual and sensorimotor skills. The automated data collection, analysis, and immediate reporting takes approximately 25 minutes to complete.

(<https://senaptec.com/sensory-station/>)

**Appendix 2 – Stroboscopic Glasses and Mobile Training App**

The Senaptec Strobe glasses and sensory training application are designed to train the connections between an individual’s eyes, brain and body.

The mobile application has 15 touch-screen visual training games related to visuo-motor, visual perception, go/no-go, depth perception, tracking etc. These can be done with or without the Strobe glasses.


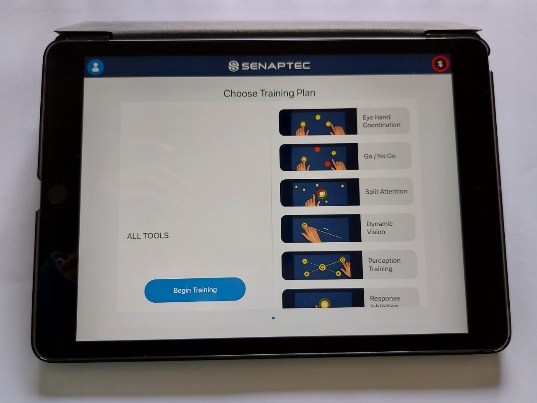


The Strobe glasses can connect to a smartphone app via Bluetooth so researchers can adjust the difficulty settings during training. Alternatively, the glasses have buttons on the side so participants can make changes themselves if required.

Liquid crystal technology lenses allow the glasses to flicker between clear and opaque, thereby removing visual information and forcing the individual to process more efficiently.

(<https://senaptec.com/>)


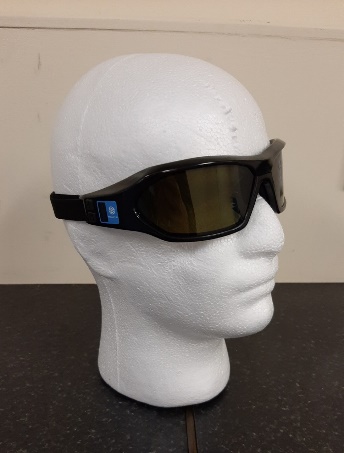


**APPENDIX 3 - App-based Visual Training Drills**

The Senaptec App has a variety drills to improve visuo-motor skills and can be run on any mobile device. As individuals improve, training adjusts automatically to remain challenging and promote continuous improvement. Examples of the type of training drills that will be used for this study are:

- **Eye Hand Coordination** - Find and touch the targets quickly and accurately.
- **Go / No-Go** - Touch the “Go” targets before they disappear, but do not touch the “No-Go” target. This requires quick decision making and swift movement.
- **Dynamic Vision** - Move your eyes to follow the target on the screen. This trains peripheral vision awareness and rapid eye movement.
- **Perception Training** - Symbols appear briefly, requiring the user to quickly see information in both central and peripheral vision. This can help the brain’s data collection and memory.
- **Response Inhibition** - Go targets and no-go targets appear momentarily in a single location. Touch the go target before it disappears and do not touch the no-go targets. This trains rapid decision making and muscle response.
- **Spatial Memory** - A set of targets will appear momentarily on the screen. You touch the screen in the locations the targets had appeared to work on memory skills.
- **Spatial Sequence** - A variation of spatial memory. In this module targets will appear and disappear sequentially. Touch the screen in the location and order the targets appeared.
- **Multiple Object Tracking** - The MOT skill often helps avoid collisions. Move a target to avoid collisions with multiple objects that are moving around the screen.
- **Depth Perception** - This module challenges and trains while the user stands at distance from the screen to make judgments of depth. Judging depth is fundamental for perceiving the world.
- **Near Far Shift** - This module uses the Senaptec handheld remote and a screen at distance to train the eye muscles to rapidly shift focus and recognize details. Quick near far shift capability is important in all areas of life, from sports to driving to walking through a store.
- **Visual Search** - Quickly find and touch the single target amongst the distractors. Search and find tasks are critical for navigating most sport and real-world activities.
- **Tempo** - Maintain rhythm by touching the target with each beat. This module is useful for training audio/visual correlation and anticipation timing.
- **Shape Cancellation** - Find and touch all the targets but do not touch the distractors. This training also serves as an assessment for visual neglect issues.
- **Visual Motor Integration** - Draw on the touch screen with your finger to repeat the pattern, either by tracing or looking at an adjacent shape. This training works on neuromotor skills.

Drills taken from: https://www.senaptec.com/product/senaptec-app

**Appendix 4 – Standard care (Active Control Interventions)**

The active control interventions will include tasks, drills and modified games that are currently used in clinical practice. Below are some examples of the type of activities that participants will undertake during the active control phase of the study.

**Appendix 5 – Feasibility Outcomes**

| Outcome | Description | Analysis |
| --- | --- | --- |
| Safety | Adverse events (mild, moderate, or severe) caused by the intervention | Total number; compare differences in adverse events between the 2 arms. |
| Amount | Duration of actual visual training undertaken during each visit | Compare average duration of visual training tolerated during each one hour visit [min] |
| Compliance/  adherence | Rate of scheduled and completed sessions | Total n sessions completed /total n sessions scheduled (%) |
| Acceptability | Motivation  Useability | Pittsburg Rehabilitation Participation Scale Scores (compare mean scores between arms)  System Usability Scale |
| Equipment | Function of mobile technology in participants’ homes | Number of issues with equipment, such as break downs, shortage of equipment, internet access |

**Appendix 6 – Safety Reporting Procedures for Adverse Events**

**(Health Research Authority Guidance on Safety Reporting, Updated July 2020)**

An adverse event (AE) is any

- This includes events related to the intervention.

- This includes events related to the procedures involved (any procedure in the intervention).

Serious Adverse Events:

A Serious Adverse Event (SAE) is defined according to the health Research Authority (HRA) Guidance dated December 2010 as, any adverse event that:

- Results in death,
- Is life-threatening illness or injury, defined as an event in which the patient is at risk of death at the time of the event,
- Requires in-patient hospitalization or prolongation of existing in-patient hospitalization,
- Results in medical or surgical intervention to prevent life threatening illness or injury or permanent impairment to a body structure or a body function, or
- Results in persistent or significant disability[^*^](https://ukc-word-edit.officeapps.live.com/we/wordeditorframe.aspx?ui=en-us&rs=en-us&wopisrc=https%3A%2F%2Flivenorthumbriaac.sharepoint.com%2Fsites%2FWeeklyPhDSupervision%2F_vti_bin%2Fwopi.ashx%2Ffiles%2Fa7eb0e34f5244e38b01ca9b22aaa5fa3&wdenableroaming=1&mscc=1&hid=6446ae67-fcce-0cf1-45b8-5a4e6a48d61d-6877&uiembed=1&uih=teams&hhdr=1&dchat=1&sc=%7B%22pmo%22%3A%22https%3A%2F%2Fteams.microsoft.com%22%2C%22pmshare%22%3Atrue%2C%22surl%22%3A%22%22%2C%22curl%22%3A%22%22%2C%22vurl%22%3A%22%22%2C%22eurl%22%3A%22https%3A%2F%2Fteams.microsoft.com%2Ffiles%2Fapps%2Fcom.microsoft.teams.files%2Ffiles%2F98076398%2Fopen%3Fagent%3Dpostmessage%26objectUrl%3Dhttps%253A%252F%252Flivenorthumbriaac.sharepoint.com%252Fsites%252FWeeklyPhDSupervision%252FShared%2520Documents%252FGeneral%252FDRAFT%2520STUDY%2520PROTOCOL_3%2520-%2520November%25202020.docx%26fileId%3Da7eb0e34-f524-4e38-b01c-a9b22aaa5fa3%26fileType%3Ddocx%26ctx%3Dfiles%26scenarioId%3D6877%26locale%3Den-us%26theme%3Ddefault%26version%3D20201007007%26setting%3Dring.id%3Ageneral%26setting%3DcreatedTime%3A1605520901066%22%7D&wdorigin=TEAMS-ELECTRON.teams.files&wdhostclicktime=1605520900939&jsapi=1&jsapiver=v1&newsession=1&corrid=33476597-f915-44e5-b22e-bbbe030fa3e1&usid=33476597-f915-44e5-b22e-bbbe030fa3e1&sftc=1&sams=1&accloop=1&sdr=6&scnd=1&hbcv=1&htv=1&hodflp=1&instantedit=1&wopicomplete=1&wdredirectionreason=Unified_SingleFlush&rct=Medium&ctp=LeastProtected#_ftn1)/incapacity, or a permanent impairment of a body function or permanent damage to a body structure.

[^*^](https://ukc-word-edit.officeapps.live.com/we/wordeditorframe.aspx?ui=en-us&rs=en-us&wopisrc=https%3A%2F%2Flivenorthumbriaac.sharepoint.com%2Fsites%2FWeeklyPhDSupervision%2F_vti_bin%2Fwopi.ashx%2Ffiles%2Fa7eb0e34f5244e38b01ca9b22aaa5fa3&wdenableroaming=1&mscc=1&hid=6446ae67-fcce-0cf1-45b8-5a4e6a48d61d-6877&uiembed=1&uih=teams&hhdr=1&dchat=1&sc=%7B%22pmo%22%3A%22https%3A%2F%2Fteams.microsoft.com%22%2C%22pmshare%22%3Atrue%2C%22surl%22%3A%22%22%2C%22curl%22%3A%22%22%2C%22vurl%22%3A%22%22%2C%22eurl%22%3A%22https%3A%2F%2Fteams.microsoft.com%2Ffiles%2Fapps%2Fcom.microsoft.teams.files%2Ffiles%2F98076398%2Fopen%3Fagent%3Dpostmessage%26objectUrl%3Dhttps%253A%252F%252Flivenorthumbriaac.sharepoint.com%252Fsites%252FWeeklyPhDSupervision%252FShared%2520Documents%252FGeneral%252FDRAFT%2520STUDY%2520PROTOCOL_3%2520-%2520November%25202020.docx%26fileId%3Da7eb0e34-f524-4e38-b01c-a9b22aaa5fa3%26fileType%3Ddocx%26ctx%3Dfiles%26scenarioId%3D6877%26locale%3Den-us%26theme%3Ddefault%26version%3D20201007007%26setting%3Dring.id%3Ageneral%26setting%3DcreatedTime%3A1605520901066%22%7D&wdorigin=TEAMS-ELECTRON.teams.files&wdhostclicktime=1605520900939&jsapi=1&jsapiver=v1&newsession=1&corrid=33476597-f915-44e5-b22e-bbbe030fa3e1&usid=33476597-f915-44e5-b22e-bbbe030fa3e1&sftc=1&sams=1&accloop=1&sdr=6&scnd=1&hbcv=1&htv=1&hodflp=1&instantedit=1&wopicomplete=1&wdredirectionreason=Unified_SingleFlush&rct=Medium&ctp=LeastProtected#_ftnref1)Disability is defined as a substantial disruption of a person’s ability to conduct normal life functions.

*Severity of an Adverse Event*

Mild Adverse Event

A mild adverse event is one that the symptoms are barely noticeable to the patient. It does not influence performance, require drug treatment or prevent the patient from carrying on with normal life activities.

Moderate Adverse Event

A moderate adverse event is one that the symptoms make the patient uncomfortable and causes some impairment to normal life activities. Treatment for symptom(s) may be required.

Severe Adverse Event

A severe event is one that the symptoms cause severe discomfort to the patient and the severity limits the patient’s normal life activities. Treatment of symptom(s) may be required.

Ref. [Safety reporting - Health Research Authority (hra.nhs.uk)](https://www.hra.nhs.uk/approvals-amendments/managing-your-approval/safety-reporting/)
