# Supplementary material for: Exploring the feasibility of technological visuo-cognitive training in Parkinson’s: Study protocol for a pilot randomised controlled trial
Source: PLoS One. 2022 Oct 7;17(10):e0275738. doi: 10.1371/journal.pone.0275738 (PMC9543984; doi:10.1371/journal.pone.0275738)
Supplement: Attachment — Submitted filename: Response to reviewers.docx [file pone.0275738.s006.docx]

We would like to thank the editors and the reviewers for their hard work in reviewing our manuscript. We have added our reviewer responses to the table below and highlighted in yellow the changes made within the manuscript.

Reviewer #1: Comments: This small RCT addresses a gap in understanding and care and has the potential to contribute to the literature and body of knowledge in the field.

| Reviewer comment | Response |
| --- | --- |
| This is a feasibility pilot study to ‘provide preliminary data to support a larger, multi-centre randomised 46 controlled trial’. As such, the main outcomes (not primary, which should be reserved for a full scale efficacy/effectiveness trial) should be the feasibility/acceptability elements and the other efficacy outcomes (i.e. TMT) should be exploratory at best. Feasibility issues such as recruitment and attrition/retention should be included and at the forefront. | The authors thank the reviewer for their advice and have made changes to the manuscript to better reflect the feasibility/acceptability outcomes as being the main focus of the study. These changes are highlighted in yellow throughout in the manuscript and summarised in the following paragraph:  “This manuscript describes the protocol that will be used to explore the feasibility of the TVT intervention as a potential rehabilitation tool in PwP by (1) evaluating the study design and procedures, (2) analysing the acceptability of the intervention by participants and (3) determining what preliminary information can be obtained to explore the efficacy of TVT to inform future trials” |
| What is the rationale for the TMT as the main efficacy outcome? | We acknowledge that the intervention has the potential to affect several different visual/motor/cognitive outcomes and we have therefore included a number of secondary outcome measures in addition to the TMT.  The TMT is widely used in clinical practice and in research to examine visuo-cognition (specifically visual attention). The authors selected the TMT as this best captures the components of visuo-cognitive function that were being targeted by the technological visuo-cognitive training intervention, and the areas of visuo-cognitive function that are known to be impaired in Parkinson’s disease, specifically visual attention.  We have added the following text in the manuscript to clarify this:  “The TMT is a standardised assessment of the components of visuo-cognitive function that were being targeted by the technological visuo-cognitive training and that are known to be impaired in PwP, specifically visual attention and processing speed. Studies have shown that impairments in attentional capacity have a significant influence on balance and gait performance in PwP (16, 78, 79). Not only is the TMT a good measure of overall cognitive function, it has also been shown to be a strong independent predictor of mobility impairment (80). |
| Is this study addressing a priority area of research/clinical care stated by the Parkinson’s community? If so, it should be stated. | Thank you for suggesting this. Parkinson’s UK have identified the primary research priorities among people with Parkinson’s, their families and professionals include research into mild thinking and memory problems, as well as balance and falls (our secondary outcomes). This study addresses these areas and we have revised the manuscript to reflect this:  “The interventions in this study have potential to address visuo-cognitive problems, balance and falls which are aligned with Parkinson’s UK top ten research priority areas”  We also carried out Patient and Public Involvement (PPI) as part of the planning for this study and have included a paragraph in the manuscript to highlight this:  “The development and design of the visuo-training protocol and documentation for this study have involved consultation with six PwP through an online focus group conducted via Zoom in October 2020. Their opinions and preferences validated the relevance of the research concept and influenced the planned length and frequency of the study interventions.” |
| The rationale is ‘novel non-pharmacological rehabilitation strategy for prevention of falls in PwP’ but number and type of falls is not being measured. The overarching aim and outcomes need to be correctly aligned.  It is not clear why there is such an emphasis on the impact on visuo-cognitive function and falls? While this may certainly be an issue, the other functional impacts appear to be ignored – what about other ADLs, driving, using eating utensils, reading etc. The study is designed as a falls’ prevention program, yet the mechanism is broader, and the main outcome addresses visual attention (i.e. TMT) – this does not align correctly. The link between falls, visuo-cognition and visual attention need to be much better explained. The manuscript would benefit from a clearly thought through logic model. | Thank you for highlighting this as an issue.  This is a feasibility study that is focussed on a novel technology for visuo-cognitive training in Parkinson’s, which aimed to examine whether it is feasible to use these technologies and to provide some initial efficacy results on improvement in visuo-cognitive function (to be used for future sample size calculations for larger studies). The study does not aim to improve falls.  We agree that improvements in visuo-cognitive function *may* contribute to improved balance and reduced falls risk in people with Parkinson’s, but this was not an aim of the current study. We have removed the reference to reducing falls in the introduction and hope that the aims and outcomes are now more clearly aligned in the revised manuscript.  “This pilot study will focus on the feasibility and acceptability of TVT in PwP and provide preliminary data to support the design of a larger, multi-centre randomised controlled trial.”  Regarding the impact of visuo-cognitive training on other functional aspects such as ADLs etc, we acknowledge that it may well have an impact and a number of different secondary outcome measures are included to capture these changes. A full list of outcomes can be viewed in Figure 1. |
| The study compares two visuo-cognitive intervention groups (technological or standard); what is the rationale that one intervention will improve TMT more than the other? Surely the approaches differ only in mode of delivery/implementation rather than mechanism of action. Thus, the focus should be on adherence/acceptability/usability etc rather than a signal of change in cognitive outcome. | The authors thank the reviewer and agree with their comments – the focus of this study is on feasibility rather than effectiveness at this stage and changes have been made throughout the manuscript to better reflect this (as outlined above).  The key difference between the two intervention groups is mode of delivery, i.e. technology vs no technology. There have been no previous studies to examine visuo-cognitive training in Parkinson’s, therefore it is unclear whether standard methods (e.g. low cost pen and paper tasks etc.) can improve visuo-cognitive function, measured via TMT. To examine whether technology is feasible and necessary to improve visuo-cognitive function, we have included both a standard care (no technology) and a technology arm to the study. |
| There are no cognition-based inclusion/exclusion criteria yet the main outcome, and indeed intervention, is visuo-cognition based. What about people with PD-MCI or parkinson’s plus syndromes? | Only participants with a confirmed “clinical diagnosis of Parkinson’s by a movement disorder specialist according to UK brain bank criteria (H&Y stage I-III)” are included in this study – please refer to lines 177-178. We are therefore not accepting individuals with Parkinson’s plus syndromes.  A “clinical diagnosis of dementia or other severe cognitive impairment” (refer to line 190) are included in the exclusion criteria. We have clarified this in the manuscript:  “Participants must score a minimum of 21/30 on the Montreal Cognitive Assessment to be able to follow instructions and undertake the training with support from the researcher.” |
| What is the basis for the sample size? Has a power analysis been done? | Due to this being a pilot study, a sample size calculation has not been performed, as the initial results from this study will help to inform future sample size calculations for a larger trial.  We have clarified this in the manuscript:  “Since this is a pilot study, a sample size calculation has not been performed. A convenience sample of forty community-living ambulatory older adults with mild-to-moderate Parkinson’s disease will participate in this study in order to explore the practicalities of delivering the interventions in relation to the feasibility parameters outlined below” |
| What assessment will be done to ascertain probability of unblinding? | Please refer to line 206-213. Due to the nature of the interventions, blinding will not be possible for participants or for the physiotherapists delivering the interventions. Two researchers are always present during the post-intervention assessments and one of those researchers will be blinded to the intervention group and will therefore carry out the assessments. We accept that there is a chance that participants may accidently reveal their group allocation to the blinded researcher and if this is observed, a third researcher may be called upon to review videos of the assessments that have taken place to ensure unbiased scoring. |
| What is the rationale and evidence for the dose of the intervention (duration, frequency, number of weeks etc)? | As this is a novel intervention in Parkinson’s, there is no gold standard with which to base the duration, frequency and length of the visuo-cognitive intervention.  In order to determine intervention dosage we initially examined evidence from stroboscopic visuo-cognitive training literature, as well as previous exercise and cognitive rehabilitation studies, with most studies delivering 2-3 1hour sessions of intervention over 4-8 weeks. We then discussed the intervention dosage with our Parkinson’s patient PPI focus group, who suggested that a 4 week intervention was more realistic for people with Parkinson’s. We were also keen to trial an intervention that could be generalisable to current (NHS) clinical practices and provision-capacity within community rehabilitation services, which typically involves interventions of similar dosage.  It may be possible that greater dosage may lead to more benefits, but due to this being the first time that a visuo-cognitive intervention, and this new technology, had been used in Parkinson’s, we took a cautious approach to the dosage. |

Reviewer #2: This is a well-written, well-considered proposal for a pilot project. I think the protocol for persons with PD may be long but the authors have considered fatigue as a barrier. Perhaps specifying eye fatigue would be helpful as this is likely to occur in this population. I have no specific edits or recommendations beyond that and recommend accepting without revision.

Our thanks to reviewer #2. We are aware that the length of the testing protocol is long and we discussed this in length during our PPI focus group work at the start of the study and during the NHS Ethics Panel review meeting. Our rationale for including so much in the protocol is that we do not know what (if any) effect the technological visuo-cognitive training will have on PwP. We therefore wanted to ensure that we have testing to cover potential changes in aspects of vision, cognition, motor function and self-efficacy in order to ensure that we do not miss any potential intervention outcomes. We have factored in regular rests, and alternate between written and physical tasks so as to reduce the risk of eye fatigue on participants. The risk of fatigue is highlighted in our Participant Information booklet and was felt to be acceptable by our PPI group participants and the Ethics Panel.
